# Relation between sleep disorders and post-stroke cognitive impairment

**DOI:** 10.3389/fnagi.2023.1036994

**Published:** 2023-07-21

**Authors:** Yajing Zhang, Xiaoshuang Xia, Ting Zhang, Chao Zhang, Ran Liu, Yun Yang, Shuling Liu, Xin Li, Wei Yue

**Affiliations:** ^1^Department of Neurology, Tianjin Huanhu Hospital, Tianjin, China; ^2^Department of Neurology, The Second Hospital of Tianjin Medical University, Tianjin, China

**Keywords:** sleep disorders, post-stroke cognitive impairment, nocturnal total sleep time, sleepiness, OSA

## Abstract

**Objective:**

To investigate the effects of sleep disorders on post-stroke cognitive impairment (PSCI) and other factors affecting post-stroke cognitive impairment.

**Methods:**

A total of 1,542 first-ever stroke inpatients in department of neurology of Tianjin Huanhu Hospital from 2015.6.1 to 2016.12.31. We recorded the personal history of patients. The MMSE (mini-mental state examination), MoCA (Montreal Cognitive Assessment), HAMD (Hamilton Depression Scale), BI (Barthel index), mRS (Modified Rankin Scale), PSQI (Pittsburgh Sleep Quality Index), ESS (Epworth Sleepiness Scale), Berlin questionnaire, nocturnal TST (total sleep time) were assessed before discharge. All patients were followed up at 3 months, 6 months, and 4 years (2019–2020) after stroke. During follow-up, the above scales should be evaluated again to assess the sleep status and cognitive function of patients at that time.

**Results:**

Nocturnal TST (>8 h) (OR 3.540, 95% CI 1.692–7.406, *P* = 0.001) was a risk factor for cognitive impairment 3 months after stroke. Nocturnal TST (<7 h) (OR 6.504, 95% CI 3.404–12.427, *P* < 0.001) was a risk factor for cognitive impairment 6 months after stroke. Low sleep quality (OR 2.079, 95% CI 1.177–3.672, *P* = 0.012), sleepiness (OR 3.988, 95% CI 1.804–8.818, *P* = 0.001), nocturnal TST (<7 h) (OR 11.334, 95% CI 6.365–20.183, *P* < 0.001), nocturnal TST (>8 h) (OR 4.096, 95% CI 1.682–9.975, *P* = 0.002) were risk factors for cognitive impairment 4 years after stroke. The prevalence of cognitive impairment with TIA were 79.3% at admission, 68.1% at 3-months follow-up, 62.1% at 6-months follow-up and 52.2% at 4-year follow-up.

**Conclusion:**

Long or short nocturnal TST (<7 h or >8 h) was a risk factor for cognitive impairment after stroke (3 months, 6 months and 4 years). Poor sleep quality and sleepiness were shown to be risk factors for cognitive impairment at 4-year follow-up. Cognitive impairment was very common in patients with TIA.

## Introduction

Stroke can lead to death and disability. The number of stroke cases is increasing globally, as is the number of post-stroke disabilities. While current research on stroke has focused on physical disability, an important aspect of cognitive impairment in stroke survivors has been neglected. The prevalence of post-stroke cognitive impairment (PSCI) will increase in the future as the incidence of stroke increases and the mortality in the elderly declines. PSCI cannot only cause physical disability, but also reduce the quality of life. As a result, PSCI has become one of the most serious public health problems in the world. Due to the high prevalence of stroke, it is necessary to assess the risk of PSCI in stroke patients so that preventive measures and rehabilitation can be targeted at high-risk groups. The direct effect of stroke events on cognition remains unclear. After stroke, the damage to the body tends to improve more or less, however, for unknown reasons, the cognitive impairment gradually worsened.

Sleep is a modifiable factor, and sleep disorders are common in elderly stroke patients. There is more and more evidence that stroke and sleep are correlated. Stroke can damage the central nervous system, leading to changes in brain activity, function and sleep structures. Sleep is regulated by a number of complex interacting mechanisms located in the brain stem, hypothalamus, preoptic region and thalamus (Scullin and Bliwise, 2015), so 20–40% of stroke patients will have sleep disorders, and 50–70% of stroke patients will have sleep-related breathing disorders. The current focus is on improving sleep in stroke patients.

Sleep disorders are associated with inflammation, metabolism, and vascular disease, affecting about 5% of the Chinese population (Ng et al., 2015). In post-stroke patients, the following disorders can be observed: sleep-disordered breathing, insomnia, sleepiness, and circadian rhythm disturbances. Sleep disorders are underdiagnosed in China. Increasing evidence also suggests that sleep is associated with cognitive function (Yaffe et al., 2014). Sleep disorders also play a role in the development of cognitive impairment, and poor sleep is a risk factor for dementia (Landry and Liu-Ambrose, 2014). The purpose of this study was to investigate sleep related factors and potential other risk factors in patients with post-stroke cognitive impairment (PSCI).

### Research object

A total of 1,542 first-ever stroke in patients, including cerebral infarction, TIA and cerebral hemorrhage, were hospitalized in department of neurology of Tianjin Huanhu Hospital from 2015.6.1 to 2016.12.31. The patients were all admitted within 72 h after onset. (1) Inclusion criteria: ➀ According to international diagnostic standards (Easton et al., 2009; Jauch et al., 2013; Hemphill et al., 2015), patients were diagnosed with cerebral infarction, TIA, and cerebral hemorrhage.➁ Patients who can provide written informed consent and are willing to follow the 4-year follow-up protocol. (2) Exclusion criteria: ➀ Patients with obvious liver and kidney dysfunction, heart failure, specific genetic diseases, severe infection, malignant disease or brain disease including cerebral infarction, cerebral hemorrhage, intracranial tumor, severe head trauma, or neurosurgery; ➁ Patients previously diagnosed with cognitive impairment. The cognitive status of patients before stroke was assessed by asking their close relatives through the Chinese version of Informant Questionnaire on Cognitive Decline in the Elderly (IQCODE), which had previously been validated in the Chinese population (Fuh et al., 1995), in order to exclude patients with pre-stroke cognitive impairment. ➂ Patients under 18 years of age; ➃ Patients with aphasia, apraxia, disturbance of consciousness, visual and hearing impairment and other reasons who have difficulty to perform functional tests and cannot accurately provide reliable information.

## Research method

All enrolled patients underwent a detailed medical history, prior history, and personal history, as well as a detailed neurological examination, magnetic resonance imaging (MRI), transcranial doppler, cervical ultrasound, magnetic resonance angiography (MRA), or CT angiography (CTA).

According to MRI results, the lesion of the patients were divided into dominant hemisphere and non-dominant hemisphere; large lesion, brain stem lesion, critical sites lesion and other lesions; multiple infarcts and non-multiple infarcts; microbleeds and non-microbleeds.

Medial temporal lobe atrophy Rating Scale (MTA) and Fazekas scale were performed. Homocysteine (Hcy), fasting blood glucose (FBG), triglycerides (TG), cholesterol (TC), high density lipoprotein (HDL), and low density lipoprotein (LDL) were recorded.

The educational level of the patients was recorded in detail. Patients with less than 6 years of education (illiterate and primary school) were in the low level of education group, and those with more than 6 years of education were in the high level of education group.

Patients were given the detailed mini-mental state examination (MMSE) score, Montreal Cognitive Assessment (MoCA score), Hamilton Depression Scale (HADM) score, National Institutes of Health Stroke Scale (NIHSS) score, Modified Rankin Scale (mRS) score, Barthel index (BI),Pittsburgh Sleep Quality Index (PSQI) score, Epworth Sleepiness Scale (ESS) score, Berlin Questionnaire (BQ), and nocturnal total sleep time (TST) before discharge, 3 months after discharge, 6 months after discharge and 4 years after discharge (2019–2020). The end point was the end of follow-up and the secondary end point was death.

The total score of MMSE is 0–30 points, and the normal value is divided into illiteracy >17 points, primary school >20 points, junior high school and above >24 points. MMSE has good clinical value for dementia screening (Mitchell, 2009; Blackburn et al., 2011; Velayudhan et al., 2014). The total score of MoCA is 0–30 points (Nasreddine et al., 2005). Patients with less than 12 years of education added one point to their MoCA score (Nasreddine et al., 2005). Cognitive impairment was defined as MoCA < 26 because this boundary produces the optimal balance between sensitivity and specificity in detecting cognitive impairment (Nasreddine et al., 2005). The combined application of MMSE and MoCA makes up for their disadvantages and improves the accuracy of screening for cognitive impairment. PSCI includes post-stroke dementia (PSD) and post-stroke cognitive impairment non-dementia (PSCIND). The diagnosis of PSD includes cognitive decline, impairment in 2 or more cognitive areas of attentional or executive function, memory, language, and visuospatial function, affecting the patient’s daily living function, which should be independent of the impaired motor and sensory function after stroke. The diagnosis of PSCIND also includes cognitive decline, impairment in at least one of the cognitive areas of attentional or executive function, memory, language, and visuospatial function, and normal or mildly affected daily living function, which should be independent of the impaired motor and sensory function after stroke. The diagnosis of PSCI is based on the patient’s clinical presentation, combined with the MoCA and MMSE scores. The diagnosis of PSCI is made by an expert panel composed of clinically experienced neuropsychiatrists, psychologists and neurologists.

Hamilton Depression Scale score >7 is considered depression. PSQI < 5 indicates high sleep quality, and PSQI ≥ 5 indicates low sleep quality. The total score of ESS is 0–24 and an overall score of ≥10 indicates sleepiness. The Berlin Questionnaire (BQ) consists of 10 questions divided into three categories: severity of snoring, daytime sleepiness, and a history of hypertension or obesity. Patients with two or more positive categories were classified as “high-risk obstructive sleep apnea (OSA) patients,” and patients with one category of positive or asymptomatic groups were classified as “low-risk OSA patients” (Kang et al., 2013).

A total of 1,542 stroke patients were enrolled and 188 patients were lost to follow-up. Among the 188 patients, 56 patients went to other places and could not cooperate with the follow-up, 103 patients withdrew from the follow-up, and 29 patients could not be contacted because their contact information was changed.

In order to reduce the rate of lost to follow-up, patients were followed up by telephone every 3–5 months, and patients were instructed to timely inform the doctors if they changed their contact information. Patients who were unable or unwilling to the hospital for follow-up will be followed up at their homes with the consent of their families. A total of 1,354 patients, including 144 who died, completed follow-up. The mortality rate was 0.9% at 3 months after stroke, 2.0% at 6 months after stroke and 10.6% at 4 years after stroke.

All data were collected by trained neurologists and nurses through standard face-to-face questionnaires. Before the follow-up, all the investigators were given special training. The training included the purpose of the study, questionnaire management methods, questionnaire testing methods and research procedures. After the training, we conducted a strict assessment, and only those who were excellent in the assessment could become investigators. Investigators were given adequate guidance and assistance during data collection.

This study had passed the ethical approval of Tianjin Huanhu Hospital. The purpose of the study had been explained to all participants and confidentiality had been promised, and participants had been informed of their right to withdraw.

PSCI includes PSD and PSCIND. The diagnosis of PSCI, PSD, and PSCIND was based on “expert consensus on the Management of Post-stroke cognitive impairment” (Wang et al., 2021), combined with MMSE and MoCA scores. The diagnosis of PSCI, PSD, PSCIND was made by a team of clinically experienced neuropsychiatrists, psychologists and neurologists. Cognitive impairment occurring within 3–6 months after stroke was defined as early-onset cognitive impairment, and cognitive impairment occurring after 6 months after stroke was defined as late-onset cognitive impairment (Mok et al., 2017). mRS ≤ 2 indicated good prognosis of neurological function and mRS > 2 indicated poor prognosis of neurological function.

### Statistics

Statistical product and service solutions (SPSS) software (version 17.0) was used for data processing and analysis. The distribution of categorical variables is expressed as a percentage. Logistic univariate regression analysis was used to analyze the factors affecting the cognitive function of patients at 3 months, 6 months and 4 years after stroke, and binary logistic regression analysis was performed for the factors with statistical significance in Logistic univariate regression analysis (*P* < 0.05). Logistic univariate regression analysis was used to analyze the factors affecting the early and late onset cognitive impairment, and binary Logistic regression analysis was performed for statistically significant factors (*P* < 0.05).

The stepwise forward method was used to select the variables that were eventually included in the model. Among the covariables, stroke was classified into TIA, cerebral hemorrhage and cerebral infarction, with cerebral hemorrhage as the reference category. TIA and cerebral infarction were combined as ischemic cerebrovascular disease. Nocturnal TST was divided into 7–8 h, <7 h, and >8 h, with 7–8 h as the reference category (Li et al., 2018). The lesions were divided into large lesion, brainstem lesion, key sites lesion, other lesions and other lesions were used as reference.

## Results

The proportion of sleep quality in each stage after stroke was shown in Table 1. The proportion of low sleep quality was 52.5% at admission, 46.8% at 3 months after stroke, 40.9% at 6 months after stroke, and 40.2% at 4 years after stroke.

**TABLE 1:** Proportion of sleep quality in each stage after stroke.

	Low sleep quality	High sleep quality
At admission	711 (52.5%)	643 (47.5%)
3 months after stroke	627 (46.8%)	713 (53.2%)
6 months after stroke	542 (40.9%)	782 (59.1)
4 years after stroke	486 (40.2%)	724 (59.8%)

The proportion of nocturnal TST in each stage after stroke is shown in Table 2. The proportion of nocturnal TST (<7 h) was 50.8% at admission, 49.3% at 3 months after stroke, 46.6% at 6 months after stroke, and 43.9% at 4 years after stroke. The proportion of nocturnal TST (>8 h) was 13.9% at admission, 12.9% at 3 months after stroke, 7.6% at 6 months after stroke, and 12.6% at 4 years after stroke.

**TABLE 2:** Proportion of nocturnal TST in each stage after stroke.

	Nocturnal TST (<7 h)	Nocturnal TST (>8 h)	Nocturnal TST (7–8 h)
At admission	688 (50.8%)	188 (13.9%)	478 (35.3%)
3 months after stroke	661 (49.3%)	173 (12.9%)	506 (37.8%)
6 months after stroke	617 (46.6%)	101 (7.6%)	606 (45.8%)
4 years after stroke	531 (43.9%)	152 (12.6%)	527 (43.5%)

The proportion of sleepiness in each stage of stroke was shown in Table 3. The proportion of sleepiness was 21.4% at admission, 21.8% at 3 months after stroke, 22.3% at 6 months after stroke, and 21.6% at 4 years after stroke.

**TABLE 3:** Proportion of sleepiness in each stage after stroke.

	Sleepiness	Non-sleepiness
At admission	290 (21.4%)	1,064 (78.6%)
3 months after stroke	292 (21.8%)	1,048 (78.2%)
6 months after stroke	295 (22.3%)	1,029 (77.7%)
4 years after stroke	261 (21.6%)	949 (78.4%)

The proportion of OSA in each stage after stroke was shown in Table 4. The proportion of high-risk OSA was 74.7% at admission, 75.8% at 3-month follow-up, 78% at 6-month follow-up, and 77.4% at 4-year follow-up.

**TABLE 4:** Proportion of OSA in each stage after stroke.

	High-risk OSA	Low-risk OSA
At admission	1,011 (74.7%)	343 (25.3%)
3 months after stroke	1,015 (75.8%)	325 (24.2%)
6 months after stroke	1,033 (78%)	291 (22%)
4 years after stroke	937 (77.4%)	273 (22.6%)

Three months after stroke, there were 288 patients without PSCI, 1,056 patients with PSCI, and 10 patients with death. The prevalence of PSCI was 78%. Univariate and multivariate logistic analyses of cognitive impairment at 3 months after stroke are shown in Table 5 (PSCI was coded as 1, non-PSCI was coded as 0).

**TABLE 5:** Univariate and multivariate logistic analyses of cognitive impairment at 3 months after stroke.

		Univariate analysis		Multivariate analysis	
		OR (95% CI)	*p*-value	OR (95% CI)	*p*-value
Population information	Gender (male)	0.523 (0.384–0.712)	<0.001*	0.672 (0.417–1.083)	0.103
	Age	1.053 (1.039–1.067)	<0.001*	0.997 (0.974–1.020)	0.792
	Low level of education	3.143 (2.227–4.436)	<0.001*	2.075 (1.332–3.232)	0.001
Stroke type	TIA	0.348 (0.175–0.692)	0.003*	2.509 (0.816–7.715)	0.109
	Cerebral infarction	0.622 (0.340–1.137)	0.123	2.858 (1.175–6.949)	0.021
Lesion characteristics	Dominant hemisphere	1.269 (0.964–1.670)	0.089	NA	
	Large lesion	0.777 (0.378–1.597)	0.492	NA	
	Brain-stem lesion	0.934 (0.597–1.461)	0.764	NA	
	Critical sites lesion	0.959 (0.654–1.407)	0.832	NA	
	Multiple lesions	2.009 (1.480–2.727)	<0.001*	0.831 (0.556–1.241)	0.365
	MTA score	2.374 (2.007–2.807)	<0.001*	1.348 (0.972–1.871)	0.074
	Fazekas score	2.128 (1.87–2.410)	<0.001*	1.585 (1.284–1.957)	<0.001
	Microbleeds	4.752 (3.190–7.080)	<0.001*	1.043 (0.580–1.875)	0.888
Risk factors	Hypertension	1.016 (0.755–1.367)	0.918	NA	
	Coronary heart disease	1.342 (0.962–1.872)	0.083	NA	
	Diabetes	1.015 (0.761–1.353)	0.920	NA	
	Drinking	0.677 (0.519–0.884)	0.004*	0.982 (0.623–1.547)	0.936
	Smoking	0.663 (0.508–0.866)	0.003*	0.832 (0.523–1.323)	0.437
Laboratory examination	Hcy	1.000 (0.990–1.011)	0.944	NA	
	TG	0.934 (0.837–1.043)	0.224	NA	
	TC	0.993 (0.983–1.003)	0.185	NA	
	HDL	2.107 (1.184–3.752)	0.011*	1.209 (0.756–1.933)	0.428
	LDL	1.073 (0.914–1.259)	0.388	NA	
Patients status at 3 months after stroke	Depression	4.107 (2.250–7.498)	<0.001*	2.243 (1.033–4.870)	0.041
	mRS > 2	8.928 (4.651–16.949)	<0.001*	3.154 (1.416–7.023)	0.005
	Low sleep quality	3.052 (2.318–4.019)	<0.001*	0.650 (0.410–1.031)	0.067
	Sleepiness	2.011 (1.427–2.833)	<0.001*	1.433 (0.792–2.594)	0.234
	Nocturnal TST (<7 h)	8.214 (6.021–11.206)	<0.001*	0.616 (0.294–1.291)	0.199
	Nocturnal TST (>8 h)	5.157 (3.331–7.983)	<0.001*	3.540 (1.692–7.406)	0.001
	High risk OSA	2.508 (1.782–3.530)	<0.001*	1.075 (0.666–1.733)	0.768

*p < 0.05, items with statistical significance in univariate analysis were included in the multivariate logistic regression analysis.

Logistic univariate analysis showed gender, age, education, stroke type, multiple lesions, MTA score, Fazekas score, microbleeds, drinking, smoking, HDL, depression at 3 months, neurological function recovery, sleep quality, sleepiness, OSA, nocturnal TST were statistically significant (*P* < 0.05). In logistic multivariate analysis, low level of education (≤6 years) (OR 2.075, 95% CI 1.332–3.232, *P* = 0.001), cerebral infarction (OR 2.858, 95% CI 1.175–6.949, *P* = 0.021), high Fazekas score (OR 1.585, 95% CI 1.284–1.957, *P* < 0.001), depression (OR 2.243, 95% CI 1.033–4.870, *P* = 0.041), poor neurological recovery (mRS > 2) (OR 3.154, 95% CI 1.416–7.023, *P* = 0.005), nocturnal TST (>8 h) (OR 3.540, 95% CI 1.692–7.406, *P* = 0.001) were risk factors for cognitive impairment at 3 months after stroke.

Six months after stroke, there were 980 patients (72.4%) with PSCI, 354 patients (26.1%) without PSCI, and 20 dead patients (1.5%). The prevalence of PSCI was 72.4%. Univariate and multivariate logistic analyses of cognitive impairment at 6 months after stroke are shown in Table 6.

**TABLE 6:** Univariate and multivariate logistic analyses of cognitive impairment at 6 months after stroke.

		Univariate analysis		Multivariate analysis	
		OR (95% CI)	*p*-value	OR (95% CI)	*p*-value
Population information	Gender (male)	0.559 (0.422–0.739)	<0.001*	0.738 (0.378–1.441)	0.374
	Age	1.057 (1.044–1.070)	<0.001*	1.005 (0.971–1.040)	0.776
	Low level of education	3.016 (2.212–4.111)	<0.001*	1.666 (0.905–3.067)	0.101
Stroke type	TIA	0.630 (0.354–1.124)	0.118	NA	
	Cerebral infarction	1.090 (0.676–1.758)	0.725	NA	
Lesion characteristics	Dominant hemisphere	1.166 (0.905–1.502)	0.234	NA	
	Large lesion	16.534 (3.998–68.381)	<0.001*	20.112 (2.248–179.936)	0.007
	Brain-stem lesion	1.712 (1.209–2.426)	0.002*	1.851 (0.886–3.866)	0.101
	Critical sites lesion	2.672 (1.945–3.671)	<0.001*	2.383 (1.291–4.399)	0.005
	Multiple lesions	2.364 (1.772–3.154)	<0.001*	0.918 (0.506–1.667)	0.779
	MTA score	2.759 (2.343–3.249)	<0.001*	1.542 (0.966–2.463)	0.070
	Fazekas score	2.353 (2.081–2.660)	<0.001*	1.532 (1.133–2.073)	0.006
	Microbleeds	7.856 (5.163–11.953)	<0.001*	1.648 (0.702–3.868)	0.251
Risk factors	Hypertension	1.037 (0.786–1.368)	0.798	NA	
	Coronary heart disease	1.315 (0.969–1.786)	0.079	NA	
	Diabetes	1.039 (0.795–1.357)	0.780	NA	
	Drinking	0.596 (0.465–0.763)	<0.001*	0.784 (0.407–1.509)	0.466
	Smoking	0.668 (0.522–0.856)	0.001*	1.150 (0.586–2.256)	0.685
Laboratory examination	Hcy	1.003 (0.992–1.014)	0.595	NA	
	TG	0.877 (0.790–0.972)	0.013*	1.016 (0.806–1.281)	0.893
	TC	0.994 (0.983–1.004)	0.223	NA	
	HDL	1.097 (0.767–1.570)	0.611	NA	
	LDL	1.015 (0.939–1.098)	0.700	NA	
Patients status at 6 months after stroke	Depression	4.434 (2.615–7.517)	<0.001*	1.522 (0.515–4.499)	0.447
	mRS > 2	9.524 (4.975–18.182)	<0.001*	1.495 (0.522–4.280)	0.453
	Low sleep quality	5.020 (3.756–6.711)	<0.001*	1.117 (0.603–2.070)	0.724
	Sleepiness	2.599 (1.607–4.202)	<0.001*	1.188 (0.504–2.800)	0.694
	Nocturnal TST (<7 h)	12.063 (8.821–16.497)	<0.001*	6.504 (3.404–12.427)	<0.001
	Nocturnal TST (>8 h)	5.715 (3.800–8.593)	<0.001*	1.383 (0.537–3.562)	0.502
	High risk OSA	4.055 (2.902–5.667)	<0.001*	1.824 (0.918–3.626)	0.086

*p < 0.05, items with statistical significance in univariate analysis were included in the multivariate logistic regression analysis.

Logistic univariate analysis showed gender, age, education, lesion, multiple lesions, MTA score, Fazekas score, microbleeds, drinking, smoking, TG, depression at 6 months, neurological function recovery, sleep quality, sleepiness, OSA, nocturnal TST were statistically significant (*P* < 0.05).

Logistic multivariate analysis showed large lesion (OR 20.112, 95% CI 2.248–179.936, *P* = 0.007), critical lesion (OR 2.383, 95% CI 1.291–4.399, *P* = 0.005), high Fazekas score (OR 1.532, 95% CI 1.133–2.073, *P* = 0.006), nocturnal TST (<7 h) (OR 6.504, 95% CI 3.404–12.427, *P* < 0.001) were risk factors for cognitive impairment at 6 months after stroke.

Four years after stroke, there were 840 patients (62.0%) with PSCI, 370 patients (27.4%) without PSCI and 144 patients (10.6%) with death. The prevalence of PSCI was 62%. Univariate and multivariate logistic analyses of cognitive impairment at 4 years after stroke are shown in Table 7.

**TABLE 7:** Univariate and multivariate logistic analyses of cognitive impairment at 4 years after stroke.

		Univariate analysis		Multivariate analysis	
		OR (95% CI)	*p*-value	OR (95% CI)	*p*-value
Population information	Gender (male)	0.620 (0.470–0.818)	0.001*	0.777 (0.420–1.435)	0.419
	Age	0.050 (1.052–1.081)	<0.001*	1.020 (0.989–1.052)	0.217
	Low level of education	2.936 (2.160–3.989)	<0.001*	2.581 (1.490–4.470)	0.001
Stroke type	TIA	0.491 (0.274–0.880)	0.017*	0.498 (0.102–2.420)	0.387
	Cerebral infarction	1.119 (0.696–1.798)	0.643	0.908 (0.290–2.846)	0.869
Lesion characteristics	Dominant hemisphere	1.029 (0.798–1.327)	0.825	NA	
	Large lesion	3.618 (1.668–7.848)	0.001*	5.087 (1.486–17.408)	0.010
	Brain-stem lesion	1.809 (1.270–2.578)	0.001*	1.885 (1.005–3.535)	0.048
	Critical sites lesion	2.831 (2.052–3.906)	<0.001*	3.389 (1.918–5.990)	<0.001
	Multiple lesions	3.160 (2.329–4.288)	<0.001*	1.870 (1.054–3.317)	0.032
	MTA score	3.551 (2.956–4.267)	<0.001*	1.372 (0.891–2.113)	0.151
	Fazekas score	3.255 (2.796–3.789)	<0.001*	2.116 (1.584–2.827)	<0.001
	Microbleeds	15.481 (8.913–26.890)	<0.001*	2.013 (0.798–5.079)	0.138
Risk factors	Hypertension	1.176 (0.894–1.549)	0.247	NA	
	Coronary heart disease	1.224 (0.894–1.675)	0.207	NA	
	Diabetes	1.338 (1.014–1.765)	0.039*	1.171 (0.710–1.932)	0.537
	Drinking	0.570 (0.444–0.731)	<0.001*	0.760 (0.417–1.383)	0.369
	Smoking	0.666 (0.520–0.855)	0.001*	0.958 (0.531–1.728)	0.886
Laboratory examination	Hcy	1.012 (0.999–1.026)	0.073	NA	
	TG	0.829 (0.744–0.924)	0.001*	1.041 (0.840–1.290)	0.714
	TC	0.957 (0.857–1.069)	0.440	NA	
	HDL	1.016 (0.730–1.416)	0.924	NA	
	LDL	1.007 (0.987–1.027)	0.493	NA	
Patients status at 4 years after stroke	Depression	13.831 (8.447–22.647)	<0.001*	7.851 (3.314–18.599)	<0.001
	mRS > 2	9.213 (4.807–17.655)	<0.001*	0.484 (0.193–1.217)	0.123
	Low sleep quality	10.380 (7.403–14.556)	<0.001*	2.079 (1.177–3.672)	0.012
	Sleepiness	9.554 (5.824–15.675)	<0.001*	3.988 (1.804–8.818)	0.001
	Nocturnal TST (<7 h)	18.665 (13.384–26.03)	<0.001*	11.334 (6.365–20.183)	<0.001
	Nocturnal TST (>8 h)	33.556 (17.188–65.51)	<0.001*	4.096 (1.682–9.975)	0.002
	High risk OSA	4.509 (3.188–6.378)	<0.001*	1.721 (0.921–3.214)	0.089

*p < 0.05, items with statistical significance in univariate analysis were included in the multivariate logistic regression analysis.

Logistic univariate analysis showed gender, age, education, stroke type, lesion, multiple lesions, MTA score, Fazekas score, microbleeds, diabetes, drinking, smoking, TG, depression at 4 years, neurological function recovery, sleep quality, sleepiness, OSA, nocturnal TST were statistically significant (*P* < 0.05).

Logistic multivariate analysis showed that low level of education (OR 2.581, 95% CI 1.490–4.470, *P* = 0.001), large lesion (OR 5.087, 95% CI 1.486–17.408, *P* = 0.01), brain-stem lesion (OR 1.885, 95% CI 1.005–3.535, *P* = 0.048), critical lesion (OR 3.389, 95% CI 1.918–5.990, *P* < 0.001), multiple lesions (OR 1.870, 95% CI 1.054–3.317, *P* = 0.032), high Fazekas score (OR 2.116, 95% CI 1.584–2.827, *P* < 0.001), depression (OR 7.851, 95% CI 3.314–18.599, *P* < 0.001), low sleep quality (OR 2.079, 95% CI 1.177–3.672, *P* = 0.012), sleepiness (OR 3.988, 95% CI 1.804–8.818, *P* = 0.001), nocturnal TST (<7 h) (OR 11.334, 95% CI 6.365–20.183, *P* < 0.001), nocturnal TST (>8 h) (OR 4.096, 95% CI 1.682–9.975, *P* = 0.002), were risk factors for cognitive impairment 4 years after stroke.

Four years after stroke, among the 840 patients with PSCI, there were 806 patients (59.5%) with early onset cognitive impairment and 34 patients (2.5%) with late onset cognitive impairment. Univariate and multivariate logistic analyses of early and late onset cognitive impairment 4 years after stroke are shown in Table 8.

**TABLE 8:** Univariate and multivariate logistic analyses of early and late onset cognitive impairment 4 years after stroke.

		Univariate analysis		Multivariate analysis	
		OR (95% CI)	*p*-value	OR (95% CI)	*p*-value
Population information	Gender (male)	2.466 (1.009–6.028)	0.048*	2.150 (0.870–5.316)	0.097
	Age	1.005 (0.971–1.041)	0.763	NA	
	Low level of education	0.710 (0.335–1.506)	0.372	NA	
Stroke type	Ischemic cerebrovascular disease	0.583 (0.199–1.712)	0.326	NA	
Lesion characteristics	Dominant hemisphere	1.555 (0.739–3.272)	0.245	NA	
	Multiple lesions	1.103 (0.531–2.293)	0.793	NA	
	MTA score	0.719 (0.489–1.058)	0.094	NA	
	Fazekas score	0.965 (0.741–1.256)	0.792	NA	
	Microbleeds	0.695 (0.328–1.474)	0.343	NA	
Risk factors	Hypertension	1.174 (0.523–2.633)	0.698	NA	
	Coronary heart disease	1.214 (0.540–2.731)	0.640	NA	
	Diabetes	0.901 (0.424–1.912)	0.785	NA	
	Drinking	1.377 (0.692–2.741)	0.363	NA	
	Smoking	1.650 (0.821–3.313)	0.159	NA	
Laboratory examination	Hcy	1.009 (0.993–1.024)	0.264	NA	
	TG	0.847 (0.564–1.271)	0.422	NA	
	TC	0.838 (0.611–1.148)	0.270	NA	
	HDL	0.812 (0.228–2.888)	0.748	NA	
	LDL	0.840 (0.556–1.268)	0.406	NA	
Patients status at 4 years after stroke	Depression	3.607 (1.702–7.645)	0.001*	3.410 (1.513–7.689)	0.003
	mRS ≤ 2	1.156 (0.471–2.839)	0.752	NA	
	Low sleep quality	1.271 (0.620–2.604)	0.512	0.867 (0.385–1.953)	0.730
	Sleepiness	1.888 (0.947–3.762)	0.071	1.124 (0.499–2.529)	0.778
	Nocturnal TST (<7 h)	2.863 (0.661–12.390)	0.159	1.719 (0.378–7.814)	0.483
	Nocturnal TST (>8 h)	5.840 (1.285–26.547)	0.022*	3.464 (0.708–16.946)	0.125
	High risk OSA	1.712 (1.220–2.402)	0.002*	0.993 (0.442–2.230)	0.987

*p < 0.05, items with statistical significance in univariate analysis were included in the multivariate logistic regression analysis.

Logistic univariate analysis showed gender, depression at 4 years, OSA and nocturnal TST were statistically significant (*P* < 0.05). Logistic multivariate analysis showed depression (OR 3.410, 95% CI 1.513–7.689, *P* = 0.003) were risk factors for late onset cognitive impairment 4 years after stroke.

The prevalence of cognitive impairment in patients with TIA was shown in Table 9.

**TABLE 9:** Prevalence of cognitive impairment in patients with TIA.

	Non-PSCI	PSD	PSCIND
At admission	28 (20.7%)	12 (8.9%)	95 (70.4%)
3 months after TIA	43 (31.9%)	10 (7.4%)	82 (60.7%)
6 months after TIA	49 (36.8%)	10 (7.5%)	74 (55.6%)
4 years after TIA	55 (47.8%)	10 (8.7%)	50 (43.5%)

The prevalence of cognitive impairment in patients with TIA was 79.3% at admission (PSD 8.9%, PSCIND 70.4%) and 68.1% at 3-month follow-up (7.4% for PSD, 60.7% for PSCIND). The prevalence of cognitive impairment in patients with TIA was 62.1% at 6 -month follow-up (7.5% for PSD, 55.6% for PSCIND) and 52.2% at 4-year follow-up (8.7% for PSD, 43.5% for PSCIND).

## Discussion

The study showed that the proportion of poor sleep quality decreased from 52.5% at admission to 40.2% at 4-year follow-up. The proportion of nocturnal TST (<7 h) decreased from 50.8% at admission to 43.9% at 4-year follow-up. The proportion of nocturnal TST (>8 h) decreased from 13.9% at admission to 12.6% at 4-year follow-up. It showed that sleep quality and sleep duration improved over time after stroke. The proportion of sleepiness ranged from 21.4% at admission to 21.6% at 4-year follow-up, suggesting that post-stroke sleepiness leveled off over time. The proportion of high risk OSA ranged from 74.7% at admission to 77.4% at 4-year follow-up, indicating that the high risk of OSA did not improve significantly after stroke over time, but increased.

An increased incidence of sleep disorders in dementia is associated with neurodegeneration and current research is focusing on whether sleep disorders can lead to cognitive decline and dementia. In a cross-sectional study of middle-aged adults (mean age 47 ± 15 years), insomnia was associated with cognitive decline (Fortier-Brochu et al., 2012). Some studies had shown a link between insomnia and decreased brain volume, including the hippocampus, frontal and parietal gray matter. Whether insomnia is a risk factor for cognitive decline and dementia, or an early marker of these conditions, is unclear. Most studies using polysomnography (PSG) had supported the link between low sleep quality and cognitive impairment. In a cross-sectional study of older women (≥65 years), sleep disturbances detected using PSG were associated with an increased risk of cognitive impairment (Blackwell et al., 2006). Prospective studies of elderly people living in communities had shown that increased sleep fragmentation was associated with increased incidence of Alzheimer’s disease and decreased cognitive function (Lim et al., 2013). More than one third of adults sleep less or more than the 7–9 h a day, and there is a lot of evidence that sleep duration can predict cognitive function in older adults. A cross-sectional study (Schmutte et al., 2007) found that self-reported long sleep duration was associated with decreased cognitive function. Another study (Xu et al., 2011) showed that both long and short sleep duration were associated with decreased cognitive function when compared to normal sleep duration (usually 7–8 h). A study of nurses (Tworoger et al., 2006) found that older women with short sleep duration were at increased risk of cognitive impairment. These studies suggested a “U-shaped” relationship between sleep duration and cognitive function, with both short and long sleep duration increasing the risk of dementia and cognitive impairment. In this study, we found that long or short nocturnal TST (<7 h or >8 h) was a risk factor for cognitive impairment after stroke (3 months, 6 months and 4 years). Poor sleep quality was a risk factor for cognitive impairment 4 years after stroke.

Sleepiness is a common symptom in adults and sleepiness is estimated to be 20–30% of the total population (Young, 2004). Sleepiness can be caused by a variety of reasons, including sleep disordered breathing (SDB), poor sleep habits, obesity, cardiovascular disease (CVD) and depression, all of which can increase the risk of cognitive impairment. Some studies had shown an independent link between daytime sleepiness and cognitive impairment. In a cross-sectional study of elderly people (≥60 years) living in a community (Ohayon and Vecchierini, 2002), after controlling for a range of confounders, older adults with daytime sleepiness were more likely to have cognitive impairment than those without daytime sleepiness. Another cross-sectional study in older adults (Merlino et al., 2010) found that, after controlling for potential confounders, daytime sleepiness was the only sleep indicator associated with dementia. Daytime sleepiness had been linked to an increased risk of cognitive decline and dementia. In this study, sleepiness was shown to be a risk factor for cognitive impairment at 4-year follow-up. A limitation of this study was that sleepiness was measured using a self-reported questionnaire, and future studies need to combine subjective and objective sleepiness measures.

OSA is more common in older adults and men. The proportion of cognitive impairment in OSA population is high. A study on elderly women showed that OSA was associated with cognitive decline (Spira et al., 2008), and APOE4 carriers were more significant. Mild to moderate OSA was associated with cognitive decline in older men who were followed up for more than 3 years and OSA patients had an 85% increased risk of mild cognitive impairment or dementia in older women followed up for 5 years (Yaffe et al., 2011). Compared with healthy controls, OSA patients showed decreased white matter integrity and decreased gray matter volume associated with memory and executive function (Joo et al., 2010). Cohort studies in older men had also shown an association between reduced oxygen saturation and cognitive decline (Blackwell et al., 2011). In this study, no relationship was found between OSA and cognitive impairment after stroke, which was related to OSA assessment by questionnaire and objective indicators such as PSG should be used for further study.

Our study showed that other factors affecting cognitive impairment after stroke included depression, low level of education, poor neurological recovery, high Fazekas score, large lesion, brain stem lesion, key site lesion. Although PSCI has received increasing attention, its risk factors have not been fully recognized. A review of PSCI risk factors showed that age, education, history of stroke, diabetes, hypertension, stroke type, stroke focus, size and location of focus, depression, and neurological function can affect the cognitive function of stroke patients (Mohd Zulkifly et al., 2016). Depression is common among stroke patients. Up to 38% of stroke patients are depressed. Depression is a risk factor for poor prognosis after stroke and is negatively associated with cognitive function. This study showed that depression was a risk factor for cognitive impairment at 3 months and 4 years after stroke, as well as a risk factor for late onset cognitive impairment.

Cognitive reserve refers to the ability of the brain to maintain the same cognitive function in the presence of disease, which is influenced by factors such as age, education and lifestyle (such as participation in intellectual activities and regular exercise) (Ballard et al., 2003; Pendlebury, 2012). Low cognitive reserve caused by low level of education may lead to higher incidence of cognitive impairment after stroke. This study showed that low level of education was a risk factor for cognitive impairment at 3 months and 4 years after stroke. PSCI was affected by stroke site and stroke severity. Leukodystrophy is an important risk factor for early onset PSCI. Leukodystrophy can lead to disruption of fronto-subcortical connections and cholinergic fiber bundle connections (Tuladhar et al., 2016), resulting in secondary cortical or subcortical regional-specific atrophy (such as medial temporal lobe atrophy), cognitive decline, and eventually dementia.

Many patients with TIA continue to have cognitive problems long after the focal neurological symptoms have been resolved. However, the prevalence and causes of post-TIA cognitive impairment remain unclear. Because cognitive function assessments are not routine for patients with TIA, cognitive impairment is often overlooked. Studies have shown that post-TIA cognitive impairment is relatively common, with 29–68% of patients with mild cognitive impairment (MCI) and 8–22% of patients with severe cognitive impairment (van Rooij et al., 2016). The higher incidence of cognitive impairment after TIA suggests that the non-local symptoms may not be as transient as local neurological symptoms. The incidence of cognitive dysfunction after TIA is lower than that after stroke. The severity and location of stroke are the major determinants of cognitive impairment after stroke (Pendlebury and Rothwell, 2009). Whether these features apply to TIA is unclear. Ischemic impairment after TIA may be the basis of cognitive impairment. The presence of vascular risk factors in patients with TIA increases the risk of white matter progression, which is also associated with cognitive decline. In addition, anxiety, depression, and delirium following TIA may affect cognitive function.

The prevalence of cognitive impairment in patients with TIA was 79.3% at admission (PSD 8.9%, PSCIND 70.4%) and 68.1% at 3-month follow-up (7.4% for PSD, 60.7% for PSCIND). The prevalence of cognitive impairment in patients with TIA was 62.1% at 6 -month follow-up (7.5% for PSD, 55.6% for PSCIND) and 52.2% at 4-year follow-up (8.7% for PSD, 43.5% for PSCIND). This study shows that cognitive impairment is very common in patients with TIA, and it should be noted that patients with TIA have persistent cognitive impairment after the complete resolution of their neurological symptoms.

Sleep disorders are common in stroke patients and are a risk factor for cognitive impairment after stroke. The sleep-wake cycle plays an important role in brain aging, which offers a potential way to improve cognitive function. Pathological changes in cognitive impairment can be present in the brain years to decades before clinical symptoms appear and it is not clear whether sleep disorders appear as an early marker of this pathology or as a risk factor for disease initiation or progression. Further prospective studies, using structural MRI and biomarkers, are needed to document the biological changes associated with sleep disorders and to investigate the mechanism of cognitive function changes after stroke.

## Conclusion

In the 4-year follow-up of patients with stroke, it was found that sleep quality and TST were improved over time after stroke, the change of sleepiness after stroke was not obvious and the high risk of OSA did not improve significantly after stroke, but showed an upward trend. Long or short nocturnal TST (<7 h or >8 h) was a risk factor for cognitive impairment after stroke (3 months, 6 months and 4 years). Poor sleep quality was a risk factor for cognitive impairment 4 years after stroke. Sleepiness was shown to be a risk factor for cognitive impairment at 4-year follow-up. Cognitive impairment is very common in patients with TIA, and we should pay attention to cognitive impairment after TIA.

### Limitation

Sleep quality, sleepiness, and OSA were measured using questionnaires, without objective measurements, which may be potentially biased. However, large studies using objective methods such as PSG are not feasible in the general population, especially in stroke patients. The PSQI questionnaire, ESS questionnaire and Berlin questionnaire are reliable screening methods for sleep disorders. Previous studies have demonstrated that sleep parameters monitored by PSG are highly correlated with subjective assessment. Importantly, our investigators were trained to conduct comprehensive interviews with patients about their sleep status and the study also conducted longitudinal follow-up on sleep status, which may lead to more accurate sleep results.

## Data availability statement

The original contributions presented in this study are included in this article/supplementary material, further inquiries can be directed to the corresponding author.

## Author contributions

YZ: conceptualization, methodology, software, and writing—original draft preparation. XX and TZ: data curation and writing—original draft preparation. CZ: visualization and investigation. RL: supervision. YY and SL: software and validation. WY and XL: writing—review and editing. All authors contributed to the article and approved the submitted version.
